# Detection of Methicillin Resistant* Staphylococcus aureus* and Determination of Minimum Inhibitory Concentration of Vancomycin for* Staphylococcus aureus* Isolated from Pus/Wound Swab Samples of the Patients Attending a Tertiary Care Hospital in Kathmandu, Nepal

**DOI:** 10.1155/2017/2191532

**Published:** 2017-01-05

**Authors:** Raghabendra Adhikari, Narayan Dutt Pant, Sanjeev Neupane, Mukesh Neupane, Roshan Bhattarai, Sabita Bhatta, Raina Chaudhary, Binod Lekhak

**Affiliations:** ^1^Department of Microbiology, Goldengate International College, Battisputali, Kathmandu, Nepal; ^2^Department of Microbiology, Grande International Hospital, Dhapasi, Kathmandu, Nepal; ^3^Central Department of Microbiology, Tribhuvan University, Kirtipur, Kathmandu, Nepal; ^4^Department of Microbiology, Nepalese Army Institute of Health Sciences, Sanobharyang, Kathmandu, Nepal

## Abstract

The present study was conducted to evaluate the performance of cefoxitin disc diffusion method and oxacillin broth microdilution method for detection of methicillin resistant* S. aureus* (MRSA), taking presence of mecA gene as reference. In addition, inducible clindamycin resistance and beta-lactamase production were studied and minimum inhibitory concentration (MIC) of vancomycin for* S. aureus* isolates was determined. A total of 711 nonrepeated pus/wound swab samples from different anatomic locations were included in the study. The* Staphylococcus aureus *was identified on the basis of colony morphology, Gram's stain, and biochemical tests. A total of 110 (15.47%)* S. aureus *isolates were recovered, of which 39 (35.50%) isolates were identified as MRSA by cefoxitin disc diffusion method. By oxacillin broth microdilution method, 31.82% of the* Staphylococcus aureus *isolates were found to be MRSA. However, mecA gene was present in only 29.1% of the isolates. Further, beta-lactamase production was observed in 71.82% of the isolates, while inducible clindamycin resistance was found in 10% of* S. aureus *isolates. The MIC value of vancomycin for* S. aureus* ranged from 0.016 *μ*g/mL to 1 *μ*g/mL. On the basis of the absolute sensitivity (100%), both phenotypic methods could be employed for routine diagnosis of MRSA in clinical microbiology laboratory; however cefoxitin disc diffusion could be preferred over MIC method considering time and labour factor.

## 1. Introduction

Although* Staphylococcus aureus *is a commensal of humans [1], it is also a frequent cause of human infections which may become serious if caused by antimicrobial resistant strains [2]. Antibiotic resistant* S. aureus*, especially MRSA, are equally adopted to hospitals and outer environments evolving as major pathogens of public health concern [3, 4].

Shortly after the introduction of methicillin in clinical world to treat infections caused by penicillinase producing* S. aureus *in 1960, MRSA emerged and spread worldwide [5, 6]. The high rate of methicillin resistance among* Staphylococcus aureus* has resulted into the increased interest for the use of clindamycin for treatment of infections caused by* S. aureus* [7]. But recently, increasing numbers of strains of* S. aureus* are acquiring resistance toward clindamycin [7].

Vancomycin is regarded as the drug of choice for treatment of infections caused by MRSA [8]. But emergence of VISA and VRSA has been reported by many authors [8]. Further, there are reports of treatment failure of the infections caused by MRSA having MIC of vancomycin just below cutoff value [8]. High vancomycin MIC for MRSA which are susceptible to vancomycin may indicate the drug resistance to many antibiotics [8].

MRSA is resistant to entire classes of *β*-lactams including cephalosporins and carbapenems and has higher risk of development of resistance to quinolones, aminoglycosides, and macrolides [9–12].

Methicillin resistance in* S. aureus *is mediated through an altered protein called low-affinity penicillin binding protein (PBP2a). PBP2a is encoded by mecA gene which is present in chromosomal mobile genetic element called Staphylococcal cassette chromosome mec (SCCmec) [13, 14]. Due to possible association of MRSA with multiple antibiotic resistance and relatively difficult and higher cost of treatment, the accurate and rapid identification of MRSA is crucial in clinical world for timely management of the infections caused by this superbug [15]. Detection of methicillin resistance in Nepal is based on cefoxitin and oxacillin disc diffusion methods with limited reports on MIC determination and detection of mecA gene by polymerase chain reaction (PCR) [16, 17]. In present study, we evaluated the performance of cefoxitin disc diffusion and oxacillin broth microdilution methods for detection of MRSA taking presence of mecA gene as reference. Further, we also studied the rates of inducible clindamycin resistance and beta-lactamase production among the strains of* S. aureus *and we determined the minimum inhibitory concentration of vancomycin for* S. aureus* isolated from pus/wound swab samples.

## 2. Materials and Methods

### 2.1. Study Site and Population

The present study was carried out among the patients (inpatients and outpatients) attending Shree Birendra Hospital, Kathmandu, Nepal, from July 2013 to January 2014. A total of 711 nonrepeated pus/wound swab samples from different anatomic locations received from the patients for bacteriological culture were included in the study.

### 2.2. Isolation and Identification of* Staphylococcus aureus*

The specimens were inoculated on blood agar and mannitol salt agar (HiMedia laboratories private limited, India) and incubated aerobically at 37°C for 48 hours. The strains of* Staphylococcus aureus* were identified on the basis of colony morphology, Gram's stain, and different biochemical tests [18].

### 2.3. Antimicrobial Susceptibility Testing

The antimicrobial susceptibility testing was performed by modified Kirby-Bauer disc diffusion technique using Mueller-Hinton agar (HiMedia laboratories private limited, India) following Clinical and Laboratory Standards Institute (CLSI) guidelines [19]. Antibiotic discs used were ciprofloxacin (5 *μ*g), clindamycin (2 *μ*g), chloramphenicol (30 *μ*g), erythromycin (15 *μ*g), gentamicin (10 *μ*g), tetracycline (30 *μ*g), cotrimoxazole (25 *μ*g), rifampin (5 *μ*g), mupirocin (200 *μ*g), and penicillin G (10 units).

### 2.4. Detection of Strains of MRSA by Cefoxitin Disc Diffusion Method

Susceptibility of* Staphylococcus aureus* isolates to cefoxitin (30 *μ*g) was determined by modified Kirby-Bauer disc diffusion method following CLSI guidelines [19]. The strains of* Staphylococcus aureus* which were found to be resistant to cefoxitin were screened as MRSA (Table 1).

### 2.5. Determination of Minimum Inhibitory Concentrations (MICs) of Oxacillin and Vancomycin

MICs of oxacillin (Table 1) and vancomycin for all isolates of* Staphylococcus aureus* were determined by broth microdilution method as described by Andrews [20] and CLSI M07-A9 guidelines [21]. The results were interpreted according to CLSI guidelines [19]. The concentrations of oxacillin used were 0.0125 *μ*g/mL to 128 *μ*g/mL and the concentrations of vancomycin used were 0.06 *μ*g/mL to 32 *μ*g/mL.

### 2.6. Detection of *β*-Lactamase Production

*β*-lactamase production in isolated* S. aureus* was detected by iodometric method as described by Samant and Pai [22].

### 2.7. Detection of Inducible Clindamycin Resistance

Erythromycin resistant isolates were tested for inducible clindamycin resistance by *D*-test as per CLSI guidelines [19].

### 2.8. Detection of mecA Gene by Polymerase Chain Reaction (PCR)

**Figure 1:**
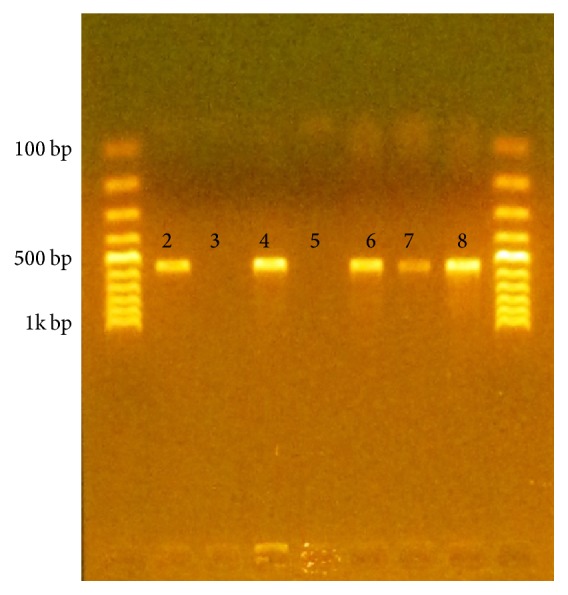
Gel electrophoresis showing the PCR products (lane 1 and lane 9: DNA ladder, lane 2: positive control, lane 3: negative control, lane 4: P18, lane 5: P36, lane 6: P53, lane 7: P78, and lane 8: P104).

Conventional phenol: chloroform method [23] was employed for extraction of chromosomal deoxyribonucleic acid (DNA) from the isolates. After optimization, the extracted DNA was subjected to PCR (Figure 1) for detection of mecA gene using PCR profiles described by Abu Shady et al. [24] (Table 1). The primer* mecA*F (5′-aaaatcgatggtaaaggttggc-3′) and the reverse primer* mecA*R (5′-agttctggagtaccggatttgc-3′) supplied by Eurogentec were used.

### 2.9. Quality Control

For quality control,* Escherichia coli *ATCC 25922,* S. aureus *ATCC 25923,* S. aureus *ATCC 29213 (mecA negative), and* S. aureus *ATCC 700699 (mecA positive) were used.

### 2.10. Data Analysis

The data obtained were analyzed with the help of statistical package for social sciences version 16.0. Chi-square test was used to analyze association between two variables and *P* value less than 0.05 was considered statistically significant.

## 3. Results

Among 711 pus/wound swab samples processed during the study, 110 (15.47%) showed culture positivity for* S. aureus*. Out of 110* S. aureus*, 39 (35.50%) isolates were MRSA by cefoxitin disc diffusion method.

### 3.1. Antibiotic Susceptibility Patterns of* S. aureus*

Among the methicillin resistant strains, highest rate of susceptibility was seen toward chloramphenicol (100%) followed by mupirocin (97.40%). Similarly, among methicillin sensitive* S. aureus* (MSSA) strains, highest rate of susceptibility was seen to rifampin and tetracycline (100%) followed by chloramphenicol and mupirocin (98.60%) (Table 2).

### 3.2. *β*-Lactamase Production among MRSA and MSSA

Beta-lactamase production was observed in 79 (71.82%) isolates of total 110* S. aureus*. Of which 52 (65.82%) isolates were MSSA and 27 (34.18%) isolates were MRSA. Statistically, there was no significant association between methicillin resistance and *β*-lactamase production (*P* value > 0.05).

### 3.3. Inducible Clindamycin Resistance among MSSA and MRSA

The inducible clindamycin resistance was observed in 11 isolates. Among which, 6 were MSSA and 5 were MRSA. Statistically, there was no significant association between methicillin resistance and inducible clindamycin resistance (*P* value > 0.05).

### 3.4. Minimum Inhibitory Concentration of Oxacillin and Vancomycin

A total of 35 (31.82%)* S. aureus *isolates were found to be MRSA by broth microdilution method with MIC cutoff value of 4 *μ*g/mL. Among them, 11 (31.43%) isolates had MIC of >128 *μ*g/mL (high level oxacillin resistant strains). The MIC of oxacillin for* S. aureus* isolates ranged from 0.032 *μ*g/mL to 256 *μ*g/mL. Only 4 out of 39 MRSA screened by cefoxitin disc diffusion method were found to be susceptible to oxacillin by broth microdilution method. Spearman's correlation between the two phenotypic methods was significant (0.922) at the 0.01 level (2-tailed). Similarly, all* S. aureus* had MIC of vancomycin below 2 *μ*g/mL (0.016 *μ*g/mL to 1 *μ*g/mL) that is susceptible to vancomycin irrespective to methicillin resistance.

### 3.5. Detection of mecA Gene

A total of 32 (29.1%)* S. aureus* isolates were found to contain mecA gene. All of the mecA containing strains of* S. aureus* were MRSA by both phenotypic methods, that is, cefoxitin disc diffusion method and oxacillin broth microdilution method. Four out of 39 MRSA screened by cefoxitin disc diffusion method, which were found to be susceptible to oxacillin by broth microdilution method, were not found to contain mecA gene. Further, the gene was found absent on MSSA detected by any of two phenotypic methods.

### 3.6. Evaluation of Cefoxitin Disc Diffusion and Oxacillin Broth Microdilution Methods in Reference to Presence of mecA Gene

MecA gene was found to be absent in 7 of the MRSA detected by cefoxitin disc diffusion method and 3 of the MRSA detected by oxacillin broth microdilution method. The sensitivity of both methods was 100% but the specificity of oxacillin broth microdilution method was greater (96.15%) than that of cefoxitin disc diffusion method (91.03%).

## 4. Discussion

In our study 35.50% of the isolates were found to be MRSA by cefoxitin disc diffusion method, which was comparable with the findings by Kshetry et al. (37.6%) [8] and Sanjana et al. (39.6%) [25]. But lower prevalence was reported by Subedi and Brahmadathan (15.4%) [26] and Baral et al. (26%) [27] and higher prevalence was reported by Khanal and Jha (68%) [16] and Tiwari et al. (69.1%) [28]. The difference in rates of isolation of MRSA in different studies might be due to the difference in locations and time periods of the studies, difference in hygienic conditions maintained in different hospitals [8], healthcare facilities provided by the hospital, implementation of infection control program, and rational use of antibiotics, which may vary from hospital to hospital [29].

No resistance of MRSA to older drug, chloramphenicol, in our study indicates routine exposure of bacteria to newly developed antibiotics and reversal of susceptibility to outdated antibiotic [30]. The low incidence of mupirocin resistance signifies low usage of the antibiotic [31].

In the present study, inducible clindamycin resistance was found in 10% of* S. aureus *isolates, which was in agreement with the result reported by Ansari et al. (12.4%) [32]. In our study, the occurrence of inducible clindamycin resistance was not significantly different among MRSA and MSSA. However, differentiation of inducible clindamycin resistant phenotypes from others is crucial for therapeutic implication of clindamycin. As use of clindamycin for treatment of the infections caused by such bacteria may result into treatment failure [7], clindamycin should not be used for treatment of such infections; rather it should be used only for the treatment of the infections caused by bacteria which are negative for inducible clindamycin resistance. Clindamycin susceptible strains which are erythromycin resistant may show inducible clindamycin resistance (*D*-test positive) and it has been suggested that inducible clindamycin resistant strains should be reported as clindamycin resistant [19]. Avoiding the use of clindamycin for the treatment of infections caused by erythromycin resistant strains also omits the chances of treatment failure [33].

In the present study, 71.1% of isolates were beta-lactamase producers by iodometric method. This is low in comparison to finding by Shrestha and Rana in nosocomial* S. aureus *isolates in Kathmandu and Lalitpur based hospitals [34]. This may be due to high rate of drug resistance among nosocomial isolates. Globally, beta-lactamase production rate lies between 55.7% and 92.6% for Staphylococci [22]. In our study, all the beta-lactamase producers were also resistant to penicillin G.

In case of MSSA, penicillin is considered superior to oxacillin to treat* S. aureus *infections if they are penicillinase nonproducers [35]. Since most of the resistance in* S. aureus *is secondary to beta-lactamase production and high level production of the enzyme results in development of borderline methicillin resistant* Staphylococcus aureus*, detection of beta-lactamase in* S. aureus *is always crucial [36].

In this study, the sensitivity of both the cefoxitin disc diffusion method and oxacillin broth microdilution method was found to be 100% but specificity of oxacillin broth microdilution method was found to be better. However, cefoxitin disc diffusion is preferred over MIC determination because it is easy to perform and requires no special equipment [37]. MecA gene was not present in some of the strains of MRSA screened by cefoxitin disc diffusion method or oxacillin broth microdilution method. But CLSI guidelines regard the isolates as MRSA if they are found resistant to either cefoxitin or oxacillin or both regardless of the presence of mecA gene [19].

Interestingly, isolates (*n* = 7) which had no mecA gene but were found to be methicillin resistant by phenotypic methods were observed to be beta-lactamase producers. Those isolates (*n* = 4) which were MRSA by cefoxitin method, but MSSA by oxacillin MIC method, had MIC value of 2 *μ*g/mL. However, the oxacillin MIC value of isolates (*n* = 3) which were MRSA by both phenotypic methods but had no mecA gene was 4 *μ*g/mL. The possible reason for methicillin resistance in absence of mecA gene may be hyperproduction of *β*-lactamase [38, 39]. Besides, in a recent study by Ballhausen et al. [40], mecC, a mecA homologue, has also been found to confer methicillin resistance in* S. aureus *in which mecA gene was absent. Though more research is needed, questions can be raised in considering mecA as sole genetic marker for methicillin resistance. But we could not check the presence of mecC as a possible reason for the phenotypic expression of methicillin resistance in absence of mecA gene. The presence of mecA gene in plasmid of* S. aureus *isolates has also been reported [41]. Since our study was completely dependent on the detection of mecA on chromosomal DNA, plasmid encoded mecA may have contributed for methicillin resistance in phenotypic tests. Therefore, all the genotypic possibilities should be analyzed for the phenotypic expression of methicillin resistance in* S. aureus *in order to discover appropriate epidemiological marker of methicillin resistance [42].

In the global scenario, 13 VRSA isolates have been isolated since its first detection in 2002 in USA with scanty reports from India and Iran [43, 44]. The vanA gene responsible for reduced susceptibility of* S. aureus *toward vancomycin has been found to be transferred from* Enterococcus faecalis *and* E. faecium* [44].

In Nepal, there are limited literatures regarding MIC of vancomycin for* S. aureus* isolated from clinical samples. We reported the MICs of vancomycin for* S. aureus* to be 0.016 *μ*g/mL to 1 *μ*g/mL. Similarly, Kshetry et al. reported the MICs of vancomycin to MRSA to be 0.125 *μ*g/mL to 1 *μ*g/mL [8]. Slightly higher MICs were reported by Amatya et al. (i.e., 0.5 *μ*g/mL to 2 *μ*g/mL) [45]. Till now no strains of* S. aureus* resistant to vancomycin have been reported from Nepal [46]. However, four VISA isolates have been reported by Pahadi et al. with MICs of vancomycin to MRSA ranging from 0.5 *μ*g/mL to 4 *μ*g/mL [46]. VISA and VRSA have been reported by many other authors from different countries [8]. Exposure of the* S. aureus* to vancomycin may be responsible for its reduced susceptibility to the reserve drug and it is attributed to the selective pressure [8]. It is difficult to treat the infections caused by VRSA due to limited antibiotics available for its treatment [8] and it is emerging as a serious public health problem.

## 5. Conclusions

On the basis of our findings, both phenotypic methods (cefoxitin disc diffusion and oxacillin broth microdilution) could be used for routine diagnosis of MRSA; however cefoxitin disc diffusion might be preferred over MIC method considering time and labour factor. MRSA and inducible clindamycin resistant* S. aureus* are emerging as a serious threat to public health in Nepal. Vancomycin can still be used as the drug of choice for treatment of infections caused by MRSA.

## Figures and Tables

**Table 1:** Comparison of the phenotypic and genotypic methods for detection of MRSA.

	Different methods used for detection of MRSA		
	Cefoxitin disc diffusion	Oxacillin broth microdilution	Polymerase chain reaction
Methods to identify MRSA strains	Strains of *S. aureus* having zone of inhibition of ≤21 mm to cefoxitin disc (30 *μ*g)	Strains of *S. aureus* having oxacillin MIC of ≥4 *μ*g/mL	Strains of *S. aureus* harboring mecA gene

**Table 2:** Antibiotic susceptibility patterns of MSSA and MRSA.

Antibiotics	MSSA	MRSA	*P* value
	Susceptible (%)	Susceptible (%)	
Erythromycin	33 (46.5)	7 (17.9)	0.003
Clindamycin	57 (80.3)	25 (64.1)	0.062
Gentamicin	64 (90.1)	14 (35.9)	0.000
Ciprofloxacin	37 (52.1)	9 (23.1)	0.003
Chloramphenicol	70 (98.6)	39 (100)	0.457
Cotrimoxazole	30 (42.2)	12 (30.8)	0.236
Mupirocin	70 (98.6)	38 (97.4)	0.664
Rifampin	71 (100)	35 (89.7)	0.006
Tetracycline	71 (100)	34 (87.2)	0.002
Penicillin G	19 (26.8)	0 (0)	0.000

## Abbreviations

MRSA: Methicillin resistant* S. aureus*
MIC: Minimum inhibitory concentration
VRSA: Vancomycin resistant* S. aureus*
VISA: Vancomycin intermediate sensitive* S. aureus*
PBP2a: Low-affinity penicillin binding protein
SCCmec: Staphylococcal cassette chromosome mec
PCR: Polymerase chain reaction
CLSI: Clinical and Laboratory Standards Institute
ATCC: American type culture collection
MSSA: Methicillin sensitive* S. aureus*
DNA: Deoxyribonucleic acid.

## References

[1] Graveland H., Duim B., van Duijkeren E., Heederik D., Wagenaar J. A. (2011). Livestock-associated methicillin-resistant *Staphylococcus aureus* in animals and humans. *International Journal of Medical Microbiology*.

[2] Monecke S., Coombs G., Shore A. C. (2011). A field guide to pandemic, epidemic and sporadic clones of methicillin-resistant *Staphylococcus aureus*. *PLoS ONE*.

[3] Gould S. W. J., Cuschieri P., Rollason J., Hilton A. C., Easmon S., Fielder M. D. (2010). The need for continued monitoring of antibiotic resistance patterns in clinical isolates of *Staphylococcus aureus*from London and Malta. *Annals of Clinical Microbiology and Antimicrobials*.

[4] Deurenberg R. H., Vink C., Kalenic S., Friedrich A. W., Bruggeman C. A., Stobberingh E. E. (2007). The molecular evolution of methicillin-resistant *Staphylococcus aureus*. *Clinical Microbiology and Infection*.

[5] Barber M. (1961). Methicillin-resistant staphylococci. *Journal of Clinical Pathology*.

[6] Chambers H. F., DeLeo F. R. (2009). Waves of resistance: *Staphylococcus aureus* in the antibiotic era. *Nature Reviews Microbiology*.

[7] Prabhu K., Rao S., Rao V. (2011). Inducible clindamycin resistance in *Staphylococcus aureus* isolated from clinical samples. *Journal of Laboratory Physicians*.

[8] Kshetry A. O., Pant N. D., Bhandari R. (2016). Minimum inhibitory concentration of vancomycin to methicillin resistant *Staphylococcus aureus* isolated from different clinical samples at a tertiary care hospital in Nepal. *Antimicrobial Resistance & Infection Control*.

[9] Baddour M. M., Abuelkheir M. M., Fatani A. J. (2006). Trends in antibiotic susceptibility patterns and epidemiology of MRSA isolates from several hospitals in Riyadh, Saudi Arabia. *Annals of Clinical Microbiology and Antimicrobials*.

[10] Koyama N., Inokoshi J., Tomoda H. (2012). Anti-infectious agents against MRSA. *Molecules*.

[11] Rehm S. J. (2008). Staphylococcus aureus: the new adventures of a legendary pathogen. *Cleveland Clinic Journal of Medicine*.

[12] Torimiro N. (2013). Analysis of Beta-lactamase production and antibiotics resistance in *Staphylococcus aureus* strains. *Journal of Infectious Diseases and Immunity*.

[13] Grundmann H., Aires-de-Sousa M., Boyce J., Tiemersma E. (2006). Emergence and resurgence of methicillin-resistant *Staphylococcus aureus* as a public threat. *The Lancet Infectious Diseases*.

[14] Ito T., Katayama Y., Asada K. (2001). Structural comparison of three types of staphylococcal cassette chromosome *mec* integrated in the chromosome in methicillin-resistant *Staphylococcus aureus*. *Antimicrobial Agents and Chemotherapy*.

[15] Johnson A. P. (2011). Methicillin-resistant *Staphylococcus aureus*: the European landscape. *Journal of Antimicrobial Chemotherapy*.

[16] Khanal L. K., Jha B. K. (2010). Prevalence of methicillin resistant *Staphylococcus aureus* (MRSA) among skin infection cases at a hospital in Chitwan, Nepal. *Nepal Medical College Journal*.

[17] Shrestha B. (2013). Comparative prevalence of MRSA in two Nepalese tertiary care hospitals. *Open Journal of Clinical Diagnostics*.

[18] Forbes B. A., Sahm D. F., Weissfeld A. S. (2007). *Bailey and Scott's Diagnostic Microbiology*.

[19] Clinical and Laboratory Standards Institute (2013). Performance standards for antimicrobial susceptibility testing: twenty third informational supplement edition. *CLSI Document*.

[20] Andrews J. M. (2001). Determination of minimum inhibitory concentrations. *Journal of Antimicrobial Chemotherapy*.

[21] Clinical and Laboratory Standardrs Institute (2012). *CLSI Document M07-A9. Methods for Dilution Antimicrobial Susceptibility Tests for Bacteria That Grow Aerobically: Approved Standard-Ninth Edition*.

[22] Samant S. A., Pai C. G. (2012). Comparative evaluation of *β*-lactamase detection methods in Staphylococci. *International Journal of Pharma and Bio Sciences*.

[23] Sambrook J., Russell D. W., Irwin N., Jansen K. A. (2001). *Molecular Cloning: A Laboratory Manual*.

[24] Abu Shady H. M., El-Essawy A. K., Salama M. S., El-Ayesh A. M. (2012). Detection and molecular characterization of vancomycin resistant *Staphylococcus aureus* from clinical isolates. *African Journal of Biotechnology*.

[25] Sanjana R., Shah R., Chaudhary N., Singh Y. (2010). Prevalence and antimicrobial susceptibility pattern of methicillin-resistant Staphylococcus aureus (MRSA) in CMS-teaching hospital: a preliminary report. *Journal of College of Medical Sciences-Nepal*.

[26] Subedi S., Brahmadathan K. N. (2005). Antimicrobial susceptibility patterns of clinical isolates of *Staphylococcus aureus* in Nepal. *Clinical Microbiology and Infection*.

[27] Baral R., Khanal B., Acharya A. (2011). Antimicrobial susceptibility patterns of clinical isolates of *Staphylococcus aureus* in Eastern Nepal. *Health Renaissance*.

[28] Tiwari H. K., Das A. K., Sapkota D., Sivarajan K., Pahwa V. K. (2009). Methicillin resistant Staphylococcus aureus: prevalence and antibiogram in a tertiary care hospital in western Nepal. *Journal of Infection in Developing Countries*.

[29] Mir B. A., Srikanth (2013). Prevalence and antimicrobial susceptibility of methicillin resistant *Staphylococcus aureus* and coagulase-negative Staphylococci in a tertiary care hospital. *Asian Journal of Pharmaceutical and Clinical Research*.

[30] Kumar A. R. (2013). Antimicrobial sensitivity pattern of *Staphylococcus aureus* isolated from pus from tertiary care hospital, Surendranagar, Gujarat and issues related to the rational selection of antimicrobials. *Scholars Journal of Applied Medical Sciences*.

[31] Dibah S., Arzanlou M., Jannati E., Shapouri R. (2014). Prevalence and antimicrobial resistance pattern of methicillin resistant *Staphylococcus aureus* (MRSA) strains isolated from clinical specimens in Ardabil, Iran. *Iranian Journal of Microbiology*.

[32] Ansari S., Nepal H. P., Gautam R. (2014). Threat of drug resistant *Staphylococcus aureus* to health in Nepal. *BMC Infectious Diseases*.

[33] Fiebelkorn K. R., Crawford S. A., McElmeel M. L., Jorgensen J. H. (2003). Practical disk diffusion method for detection of inducible clindamycin resistance in *Staphylococcus aureus* and coagulase-negative Staphylococci. *Journal of Clinical Microbiology*.

[34] Shrestha B., Rana S. (2014). Comparative study of three *β* lactamase test methods in *Staphylococcus aureus* isolated from two Nepalese hospitals. *Open Journal of Clinical Diagnostics*.

[35] Kaase M., Lenga S., Friedrich S. (2008). Comparison of phenotypic methods for penicillinase detection in *Staphylococcus aureus*. *Clinical Microbiology and Infection*.

[36] McDougal L. K., Thornsberry C. (1986). The role of *β*-lactamase in staphylococcal resistance to penicillinase-resistant penicillins and cephalosporins. *Journal of Clinical Microbiology*.

[37] Farahani A., Mohajeri P., Gholamine B., Rezaei M., Abbasi H. (2013). Comparison of different phenotypic and genotypic methods for the detection of methicillin-resistant *Staphylococcus aureus*. *North American Journal of Medical Sciences*.

[38] Boyce J. M., Medeiros A. A. (1987). Role of *β*-lactamase in expression of resistance by methicillin-resistant *Staphylococcus aureus*. *Antimicrobial Agents and Chemotherapy*.

[39] Barber M., Rozwadowska-Dowzenko M. (1948). Infection by penicillin-resistant staphylococci. *The Lancet*.

[40] Ballhausen B., Kriegeskorte A., Schleimer N., Peters G., Becker K. (2014). The *mecA* homolog *mecC* confers resistance against *β*-lactams in *Staphylococcus aureus* irrespective of the genetic strain background. *Antimicrobial Agents and Chemotherapy*.

[41] Bennimath V. D., Gavimath C. C., Kalburgi P. B., Kelmani C. (2011). Amplification and Sequencing of *mecA* gene from methicillin resistant *Staphylococcus aureus*. *International Journal of Advanced Biotechnology and Research*.

[42] Chen F.-J., Huang I.-W., Wang C.-H. (2012). *mecA*-positive *Staphylococcus aureus* with low-level oxacillin MIC in Taiwan. *Journal of Clinical Microbiology*.

[43] Gardete S., Tomasz A. (2014). Mechanisms of vancomycin resistance in *Staphylococcus aureus*. *Journal of Clinical Investigation*.

[44] Rossi F., Diaz L., Wollam A. (2014). Transferable vancomycin resistance in a community-associated MRSA lineage. *The New England Journal of Medicine*.

[45] Amatya R., Devkota P., Gautam A. (2014). Reduced susceptibility to vancomycin in methicillin resistant *Staphylococcus aureus*: a time for action. *Nepal Medical College Journal*.

[46] Pahadi P. C., Shrestha U. T., Adhikari N., Shah P. K., Amatya R. (2014). Growing resistance to vancomycin among methicillin resistant *Staphylococcus aureus* isolates from different clinical samples. *Journal of Nepal Medical Association*.

